# Troponin T Is Negatively Associated With 3 Tesla Magnetic Resonance Peripheral Nerve Perfusion in Type 2 Diabetes

**DOI:** 10.3389/fendo.2022.839774

**Published:** 2022-05-10

**Authors:** Johann M. E. Jende, Christoph Mooshage, Zoltan Kender, Lukas Schimpfle, Alexander Juerchott, Peter Nawroth, Sabine Heiland, Martin Bendszus, Stefan Kopf, Felix T. Kurz

**Affiliations:** ^1^ Department of Neuroradiology, Heidelberg University Hospital, Heidelberg, Germany; ^2^ Department of Endocrinology, Diabetology and Clinical Chemistry (Internal Medicine 1) Heidelberg University Hospital, Heidelberg, Germany; ^3^ Division of Experimental Radiology, Department of Neuroradiology, Heidelberg, Germany; ^4^ German Center of Diabetes Research, Associated Partner in the Deutsches Zentrum für Diabetesforschung (DZD), München-Neuherberg, Germany; ^5^ German Cancer Research Center, Division of Radiology, Heidelberg, Germany

**Keywords:** diabetic neuropathy, magnetic resonance neurography, dynamic contrast enhancement, perfusion, troponin T

## Abstract

**Objective:**

The pathogenesis of diabetic polyneuropathy (DN) is poorly understood and given the increasing prevalence of DN, there is a need for clinical or imaging biomarkers that quantify structural and functional nerve damage. While clinical studies have found evidence of an association between elevated levels of troponin T (hsTNT) and N-terminal pro brain natriuretic peptide (proBNP) with microvascular compromise in type 2 diabetes (T2D), their implication in mirroring DN nerve perfusion changes remains unclear. The objective of this study was, therefore, to investigate whether hsTNT and proBNP assays are associated with MRI nerve perfusion in T2D.

**Methods:**

In this prospective cross-sectional single-center case-control study, 56 participants (44 with T2D, 12 healthy control subjects) consented to undergo magnetic resonance neurography (MRN) including dynamic contrast-enhanced (DCE) perfusion imaging of the right leg. Using the extended Tofts model, primary outcome parameters that were quantified are the sciatic nerve’s microvascular permeability (K^trans^), the extravascular extracellular volume fraction (v_e_), and the plasma volume fraction (v_p_), as well as hsTNT and proBNP values from serological workup. Further secondary outcomes were clinical, serological, and electrophysiological findings.

**Results:**

In T2D patients, hsTNT was negatively correlated with K^trans^ (r=-0.38; p=0.012) and v_e_ (r=-0.30; p=0.048) but not with v_p_ (r=-0.16; p=0.294). HsTNT, K^trans^, and v_e_ were correlated with peroneal nerve conduction velocities (NCVs; r=-0.44; p=0.006, r=0.42; p=0.008, r=0.39; p=0.014), and tibial NCVs (r=-0.38;p=0.022, r=0.33; p=0.048, r=0.37; p=0.025). No such correlations were found for proBNP.

**Conclusions:**

This study is the first to find that hsTNT is correlated with a decrease of microvascular permeability and a reduced extravascular extracellular volume fraction of nerves in patients with T2D. The results indicate that hsTNT may serve as a potential marker for the assessment of nerve perfusion in future studies on DN.

## Introduction

Diabetic polyneuropathy (DN) is one of the most frequent and most disabling complications of diabetes mellitus (1). Especially in type 2 diabetes (T2D), the complex pathophysiological mechanisms that cause DN have not been understood completely (1–4). Evidence from clinical and histological studies suggests that nerve ischemia related to macro- and microangiopathy as well as cardiac insufficiency is a major contributor to demyelination and axonal damage in T2D (5–8). Recent studies have found cardiac biomarkers, high sensitivity troponin T (hsTNT) and N-terminal pro brain natriuretic peptide (proBNP), to be associated with microvascular complications in patients with T2D (9). It remains to be determined, however, whether hsTNT and proBNP are also associated with the occurrence of DN and whether both hsTNT and proBNP codify parameters of nerve perfusion such as plasma volume or microvascular permeability (9). To date, is has not been possible to assess the perfusion of peripheral nerves directly in the context of clinical studies. Magnetic resonance neurography (MRN) at 3 Tesla (3T) is a non-invasive method that allows to visualize and quantify structural and physiological changes of peripheral nerves along the entire anatomical course (10, 11). Recent studies on MRN in patients with T2D have found that structural nerve damage associated with demyelination in T2D is related to elevated levels of hsTNT but not proBNP (12). While there are several animal experimental MRI studies on the vascular supply of peripheral nerves and monitoring changes in microvasculature (13–16), only a recent pilot study on dynamic contrast enhanced (DCE) MRN in patients focusing on inflammatory neuropathies could, for the first time, demonstrate that DCE sequences allow investigating the perfusion of peripheral nerves by assessing parameters related to plasma volume and microvascular permeability (17). These parameters can be obtained from DCE MRN using the extended Tofts model, which allows calculating the constant of the examined nerve’s capillary permeability (K^trans^), the volume fraction of the plasma space (v_p_), and the volume fraction of the extracapillary extracellular space (v_e_) (18, 19). The aim of this study was to combine DCE imaging of nerves at thigh level in patients with T2D with hsTNT and proBNP assays, and demographic, clinical, and electrophysiological data in order to investigate potential associations between hsTNT and proBNP with parameters of nerve microcirculation in patients with T2D.

## Methods

### Study Design and Participants

This study was approved by the ethics committee of Heidelberg University Hospital (HEIST-DiC, clinicaltrials.gov identifier NCT03022721, local ethics number S-383/2016) and all participants gave written informed consent. Overall exclusion criteria were age <18, pregnancy, an estimated glomerular filtration rate (eGFR) <60ml/min, any contraindications for MR imaging or administration of MRI contrast agents. Further reasons for exclusion were history of myocardial infarction, coronary heart disease, heart surgery, spine surgery, lumbar disc extrusion, risk factors for sarcopenia or neuropathy other than diabetes such as malignant diseases, alcoholism, hypovitaminosis, previous or ongoing exposure to neurotoxic agents, chronic neurological diseases such as Parkinson’s disease, restless legs syndrome, or multiple sclerosis. The sample size was based on the results of previous MRN studies on DN (12, 20) and 44 patients with T2D (17 women, 27 men) and 12 controls (7 women, 5 men) were enrolled in this prospective single-center study between June 2016 and March 2020 and underwent DCE MRN with subsequent clinical, electrophysiological, and serological assessments.

### Clinical and Electrophysiological Examination

For every participant, a detailed medical history was taken. Electrophysiological examinations (VikingQuest; Viasys Healthcare GmbH, Höchberg, Germany) included an assessment of nerve conduction velocities (NCVs) of the tibial, peroneal, and sural nerve, distal motor latencies (DMLs) of the right tibial and peroneal nerve, compound muscle action potentials (CMAPs) of the tibial and peroneal nerve, and sensory nerve action potentials (SNAPs) of the sural nerve of the right leg. Skin temperature was kept at 32°C throughout the examination. Electrophysiological studies were conducted by two specially trained medical technical assistants with more than 6 years of experience in electrophysiological assessments on patients with diabetes. An examination of neuropathic symptoms was performed comprising the neuropathy disability score (NDS) and the neuropathy severity scale (NSS) (21). In line with Gibbon’s criteria for DN, patients with an NDS ≥ 3 were assigned to the DN group (22).

### MRI Imaging Protocol

All participants underwent high-resolution MRN of the right thigh in a 3.0 Tesla MR-scanner (Magnetom Tim TRIO, Siemens Healthineers, Erlangen, Germany). A 15-channel transmit-receive extremity coil was used and the following sequences were applied:

1) axial high resolution T2-weighted turbo spin echo (TSE) 2D sequence with spectral fat suppression; repetition time (TR) = 5970 ms, echo time (TE) = 55 ms, field of view (FOV) = 160 × 160 mm^2^, matrix size = 512 × 512, slice thickness = 4 mm, no interslice gap, voxel size = 0.3 × 0.3 × 4.0 mm^3^, 24 slices, 24 acquired images, total acquisition time = 4:42 min;
2) axial T1-weighted volume interpolated breathhold examination (VIBE) sequence; TR = 3.3 ms, TE = 1.11 ms, FOV = 160 × 160 mm^2^, matrix size = 128 × 128, slice thickness = 4 mm, interslice gap = 0.8 mm, voxel size = 1.3 × 1.3 × 4.0 mm^3^; single acquisition at a flip-angle of 5°, 8°, 11°, 14°, 17° (24 slices = 144 acquired images), total acquisition time = 30s;
3) axial T1-weighted volume interpolated breathhold examination (VIBE) sequence; TR = 3.3 ms, TE = 1.11 ms, FOV = 160 × 160 mm^2^, matrix size = 128 × 128, slice thickness = 4 mm, interslice gap = 0.8 mm, voxel size = 1.3 × 1.3 × 4.0 mm^3^ 50 repetitions (1200 acquired images) at a flip angle of 15°, contrast agent administration (Dotarem^®^, Guerbet, France, 0.1 mmol/kg, flow rate 3.5ml/s) after completion of the sixth repetition, total acquisition time = 4:09 min.

The sequence was centered on the sciatic nerve bifurcation at distal thigh level in every participant.

### MRI Data Analysis and Statistical Analysis

**Figure 1:**
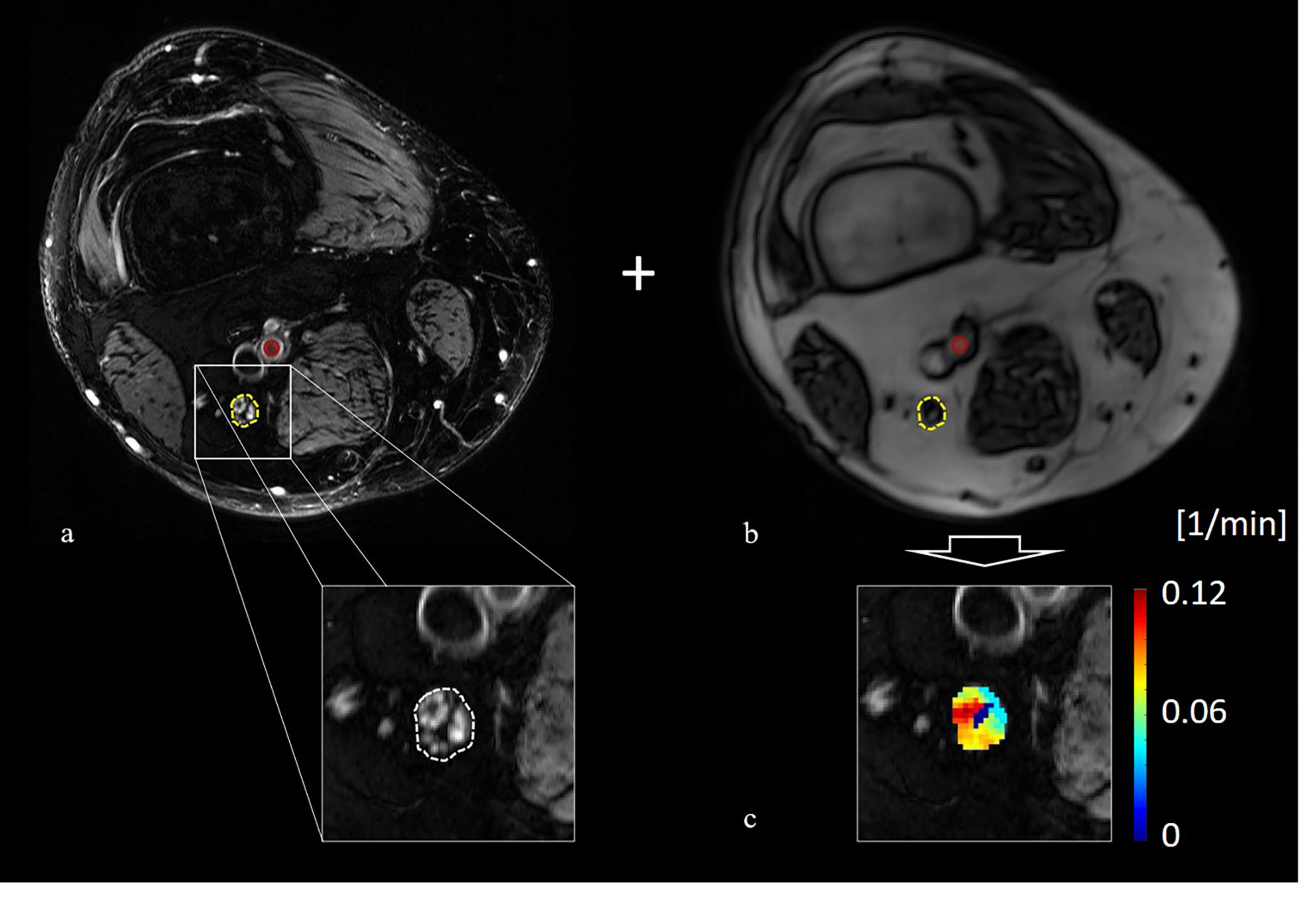
Principle of image co-registration and assessment of nerve perfusion parameters: **(A)** T2-weighted image of the distal right thigh showing the tibial compartment of the sciatic nerve (yellow circle) and the position of the femoral artery (red circle), **(B)** axial T1-weighted volume interpolated breathhold examination sequence of the same position a s in **(A)** with the co-registered position of the sciatic nerve (yellow circle) and the femoral artery (red circle), **(C)** color coded map of K^trans^ values obtained from the extended Tofts model.

All images were pseudonymized and observers were blinded to all clinical data. For each patient, we segmented the sciatic nerve manually on all images of the T2-weighted sequence with ImageJ (23). T2-weighted images were also co-registered to the T1 VIBE sequence with affine transformations using custom-written code in Matlab (MathWorks, Natick, MA, R2020b) (24). The schematic process of image segmentation and co-registration is shown in **Figure 1**. We manually determined the arterial input function (AIF) by segmenting a region of interest of the femoral artery on a representative imaging slice. The average signal intensity of all artery voxels for consecutive imaging time points was then used to obtain the signal intensity curve during contrast administration. The signal intensity baseline end was determined as the last imaging time point before signal intensity would increase by more than 25% above the averaged signal intensity of all preceding imaging time points. The resulting AIF was smoothed with a moving average filter of 3 images.

We determined the relaxation time *T*_1,0_ for the first imaging time point subsequently for each voxel in dependence on flip angle *α* by assigning *T*_1,_ *_x_* (*α*) = *SI*_0_/tan(*α*) and *T*_1,_ *_y_* (*α*) = *SI*_0_/sin(*α*), where *SI*_0_ represents the initial signal intensity. Subsequent linear regression on *T*_1,_ *_x_* and *T*_1,_ *_y_* yields the slope *m* (25). Spin-lattice relaxation time *T*_1,0_ follows as *T*_1,0_ = -*TR*/log(*m*), with repetition time *TR*. Using *α_R_* = 15°, we obtain with signal intensity *SI_t_* and relaxation time *T*_1,_ *_t_* at time *t*: $\frac{1}{T_{1,t}}=-\log(\frac{\left[1-C_{a}\right]}{1-\cos(\alpha_{R})C_{a}})/TR$ , where $C_{a}=\frac{\frac{SI_{t}}{SI_{0}}\left[1-\exp\left(-\frac{TR}{T_{1,0}}\right)\right]}{\left[1-\cos\left(\alpha_{R}\right)\exp\left(-\frac{TR}{T_{1,0}}\right)\right]}$ , c.f. Eq. (6) in (26). The tissue concentration *C(t)* at time *t* is then found as $C(t)=\frac{1}{r_{1}}[\frac{1}{{\text{T}}_{1,\text{t}}}-\frac{1}{T_{1,0}}]$ (26), where *r*_1_ = 3.43 L/mmol/s represents the relaxivity of blood at 3 Tesla (27).

We chose the extended Tofts model (ETM) which is used as a default perfusion model in central nervous system imaging and diabetes, see e.g., (19). It consists of a plasma and an extravascular extracellular compartment. Simpler perfusion models, such as the Patlak model, assume a negligible diffusion of contrast agent from the extravascular space back to the intravascular space, however, this *a priori* assumption cannot be justified in diabetes where it was shown that the permeability of the blood-brain barrier is generally increased (28). In the ETM, we have (29): $C_{M}(t)=K^{trans}\int_{0}^{t}AIF(t^{\prime })\exp\left(-\frac{K^{\text{trans}}\left[t-t^{\prime }\right]}{v_{e}}\right)dt^{\prime }+v_{p}AIF(t),$

where *K^trans^* represents the volume transfer constant between plasma and the extravascular extracellular compartment, *v_e_* represents the volume fraction of extravascular extracellular space per unit volume of tissue, *v_p_* is blood plasma volume per unit volume of tissue, and *C_M_* (*t*) corresponds to the model tissue concentration at time *t*. To minimize the residual sum of least squares, Σ*_t_* [*C_M_* (*t*) – *C*(*t*)]^2^, we used the Nelder-Mead simplex method for model parameters *K^trans^*, *v_e_*, and *v_p_* with starting values *K^trans^* = 0.007/s, *v_e_* = 0.15, *v_p_* = 0.025 (17, 30–32).

### Statistical Analysis

Statistical data analysis was carried out with MATLAB (R2020b) and GraphPad Prism 7. The D’Agostino-Pearson omnibus normality test was used to test for Gaussian normal distribution. If a Gaussian normal distribution was given, t tests were used for comparisons of two groups, one-way ANOVAs were used for comparisons of more than two groups, and Pearson correlation coefficients were used for correlation analysis. If data were not Gaussian distributed, the Mann-Whitney test was used for comparisons of two groups, the Kruskal-Wallis test with *post-hoc* Dunn correction was used for multiple comparisons of more than two groups, and nonparametric Bonferroni-corrected Spearman correlation was used for correlation analysis. In case of multiple significant correlations for one parameter, partial correlation analysis with controlling for confounding variables was performed.

## Results

### Demographic and Clinical Data

This study comprised 44 patients with T2D (17 women, 27 men, mean age 66.14 ± 7.12) and 12 controls (7 women, 5 men mean age 61.58 ± 7.79). Between T2D patients and controls, there were no significant differences for age, gender, BMI, or glomerular filtration rate. In the T2D group, 26 patients suffered from DN. On electrophysiological examination, lower tibial and peroneal NCVs and CMAPs were found in T2D patients compared to controls. A summary and comparison of demographic and clinical data of patients with T2D and controls is provided in **Table 1**.

**Table 1:** Comparison of demographic, serologic, clinical, electrophysiological, and MRN imaging data of all study participants.

	T2D	Controls	p
**K^trans^ (min^-1^)**	0.040 ± 0.011	0.035 ± 0.011	0.207^T^
**v_p_ (%)**	4.74 ± 0.82	4.63 ± 0.56	0.959^M^
**v_e_ (%)**	3.28 ± 4.58	1.64 ± 1.71	0.118 ^M^
**Age (years)**	66.14 ± 7.12	61.58 ± 7.79	0.076^T^
**Diabetes duration (years)**	10.18 ± 9.53	n.a.	n.a.
**Gender (w/m)**	17 w/27m	7w/5m	0.229^M^
**BMI (kg/m^2^)**	28.68 ± 4.11	27.47 ± 3.64	0.359^T^
**hsTNT (pg/mL)**	9.93 ± 4.04	7.25 ± 2.18	0.032^T^
**proBNP (pg/mL)**	115.30 ± 121.60	75.92 ± 52.20	0.646^M^
**HbA1c %**	6.88 ± 1.23	5.58 ± 0.55	<0.001^M^
**GFR (ml/min)**	87.55 ± 15.05	87.50 ± 13.88	0.992^T^
**NDS**	3.56 ± 3.08	1.33 ± 1.44	0.026^M^
**NSS**	4.14 ± 3.43	2.33 ± 3.60	0.144^M^
**Sural nerve NCV (m/s)**	45.13 ± 7.06	45.83 ± 4.82	0.758^M^
**Sural nerve SNAP (µV)**	5.54 ± 3.20	7.89 ± 4.71	0.070^M^
**Peroneal NCV (m/s)**	39.59 ± 5.40	45.08 ± 4.60	0.002^T^
**Peroneal CMAP (mV)**	5.02 ± 4.01	8.88 ± 6.96	0.020^M^
**Peroneal DML (ms)**	7.37 ± 13.13	3.94 ± 0.69	0.009^M^
**Tibial NCV (m/s)**	40.81 ± 5.02	44.75 ± 4.00	0.017^T^
**Tibial CMAP (mV)**	9.68 ± 6.41	16.58 ± 8.24	0.004^T^
**Tibial DML (ms)**	5.00 ± 3.01	3.62 ± 0.53	0.014^M^

All values are displayed as mean ± standard deviation. K^trans^, constant of permeability; v_p_, plasma volume fraction; v_e_, extracellular extravascular volume fraction; BMI, body-mass index; hsTNT, high sensitivity troponin T; proBNP, pro brain binatriuretic peptide; n.a., not applicable; NDS, neuropathy disability score; NSS, neuropathy severity scale; NCV, nerve comnduction velocity; CMAP, compound motor action potential; SNAP, sensory nerve action potential; m/s, meters per second; ms, miliseconds; mV, millivolts; µV, microvolts; ^M^, p value obtained from Mann-Whitney U test; ^T^, value obtained from t-Test.

### Group Comparisons of hsTNT and proBNP Levels for T2D Patients and Controls

**Figure 2:**
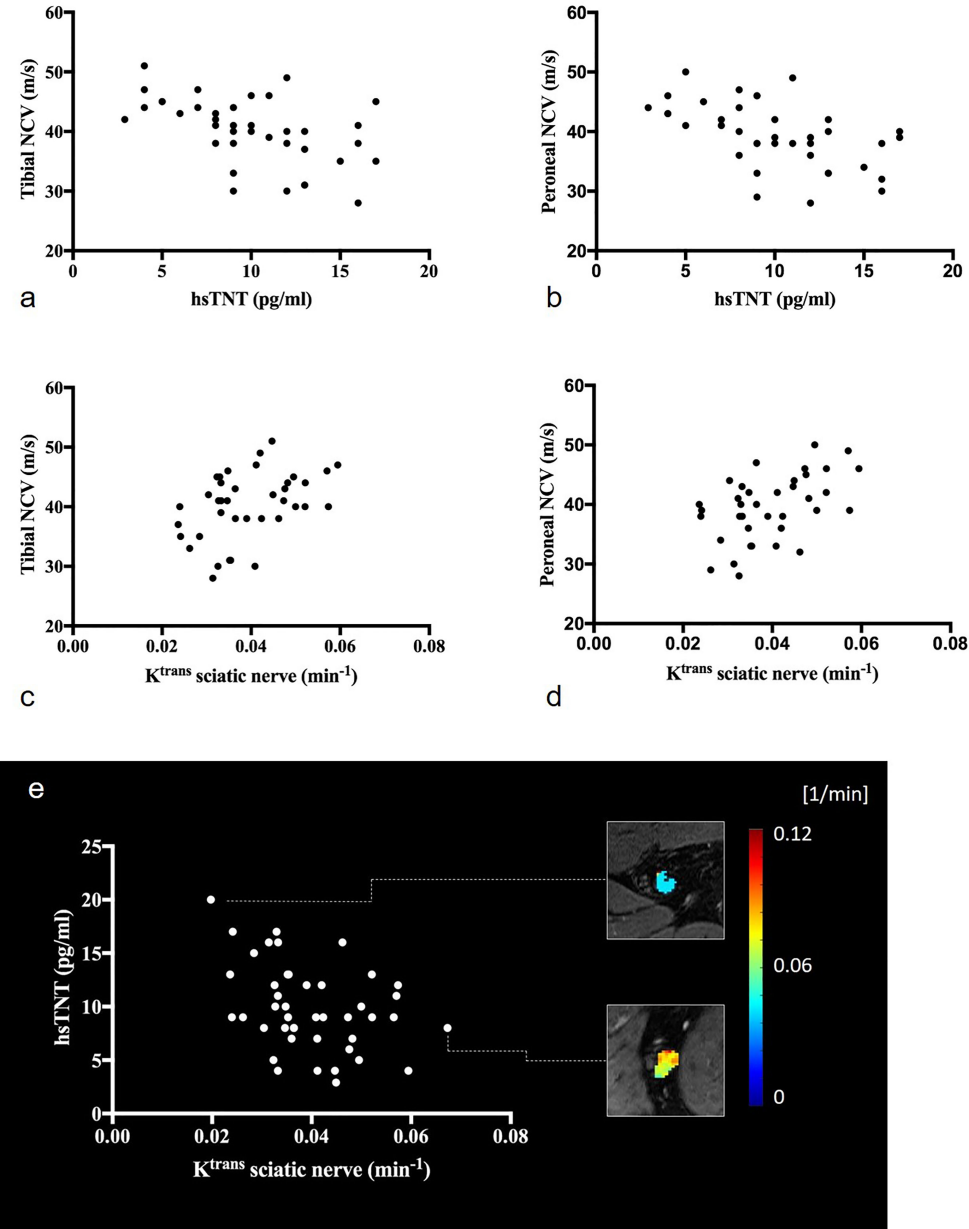
Correlations of the sciatic nerve’s K^trans^ and hsTNT with electrophysiologic parameters: **(A)** correlation of hsTNT with tibial nerve conduction velocities (r=-0.51; p=0.001), **(B)** correlation of hsTNT and peroneal nerve conduction velocities (r=-0.53; p=0.001), **(C)** correlation of K^trans^ with tibial nerve conduction velocities (r=0.42; p=0.008), **(D)** correlation of K^trans^ and peroneal nerve conduction velocities (r=0.49; p=0.002), **(E)** illustration of the correlation between K^trans^ and hsTNT (r=-0.38; p=0.012). Representative color-coded K^trans^ maps of the sciatic nerve are shown. Upper rectangle: T2D patient with hsTNT levels of 20pg/ml and a sciatic nerve’s K^trans^ of 0.020 (min^-1^), lower rectangle: T2D patient with hsTNT levels of 8pg/ml and a sciatic nerve’s K^trans^ of 0.067 (min^-1^).

Patients with T2D showed higher levels of hsTNT compared to controls (9.93 ± 0.04 pg/mL versus 7.25 ± 2.18 pg/mL, respectively, p=0.032), see **Table 1**. Also, ANOVA revealed that hsTNT was higher in T2D patients with DN compared to T2D patients without DN (11.67 ± 3.50 vs. 8.65 ± 4.05; p=0.015) and compared to controls (vs. 7.25 ± 2.18; p=0.002), no such differences were found for proBNP. In T2D patients, hsTNT correlated negatively with tibial and peroneal NCVs and with tibial CMAPs. A positive correlation was found between hsTNT and age. No such correlations were found for proBNP. A summary of all correlations of hsTNT and proBNP in T2D patients and controls is provided in **Table 2**, and correlations of hsTNT with tibial and peroneal NCVs are illustrated in **Figures 2A, B**.

**Table 2:** Correlations of hsTNT and proBNP with MRN perfusion parameters and demographic, serologic, clinical, and electrophysiological data.

	hsTNT T2D (pg/mL)		proBNP T2D (pg/mL)		hsTNT Co (pg/mL)		proBNP Co (pg/mL)	
	r	p	r	p	r	p	r	p
**K^trans^ (min^-1^)**	-0.38	0.012	-0.09	0.569	0.39	0.241	0.03	0.931
**v_p_ (%)**	-0.16	0.294	-0.17	0.280	-0.20	0.558	-0.18	0.602
**v_e_ (%)**	-0.30	0.048	-0.05	0.732	0.31	0.353	0.21	0.529
**Age (years)**	0.35	0.018	0.13	0.393	0.23	0.479	0.55	0.063
**Diabetes duration (years)**	-0.07	0.694	-0.12	0.499				
**Gender**	-0.16	0.291	0.41	0.006	-0.38	0.217	-0.23	0.463
**BMI (kg/m^2^)**	-0.03	0.824	0.17	0.274	0.06	0.860	-0.34	0.286
**hsTNT (pg/mL)**			0.10	0.503			0.62	0.032
**proBNP (pg/mL)**	0.10	0.503			0.62	0.032		
**HbA1c %**	0.24	0.120	-0.03	0.850	0.27	0.398	-0.06	0.849
**GFR (ml/min)**	-0.25	0.123	-0.24	0.137	0.33	0.295	0.31	0.325
**NDS**	0.45	0.003	0.09	0.556	0.17	0.588	-0.02	0.941
**NSS**	0.17	0.276	0.12	0.431	0.27	0.403	0.54	0.068
**Sural nerve NCV (m/s)**	-0.33	0.128	<0.01	0.986	0.00	0.989	-0.23	0.472
**Sural nerve SNAP (µV)**	-0.19	0.320	-0.16	0.398	-0.10	0.757	-0.16	0.616
**Peroneal NCV (m/s)**	-0.53	0.001	-0.03	0.850	-0.31	0.326	-0.58	0.049
**Peroneal CMAP (mV)**	-0.19	0.259	-0.18	0.284	0.15	0.637	-0.23	0.482
**Peroneal DML (ms)**	0.12	0.468	-0.27	0.101	0.31	0.330	0.47	0.121
**Tibial NCV (m/s)**	-0.51	0.001	0.254	0.124	0.22	0.500	0.04	0.905
**Tibial CMAP (mV)**	-0.55	0.001	0.19	0.276	-0.70	0.012	-0.55	0.065
**Tibial DML (ms)**	0.18	0.288	-0.04	0.823	0.44	0.155	0.55	0.064

K^trans^, constant of permeability; v_p_, plasma volume fraction; v_e_, extracellular extravascular volume fraction; BMI, body-mass index; hsTNT, high sensitivity troponin T; proBNP, pro brain binatriuretic peptide; NDS, neuropathy disability score; NSS, neuropathy severity scale; NCV, nerve comnduction velocity; CMAP, compound motor action potential; SNAP, sensory nerve action potential; m/s, meters per second; ms, miliseconds; mV, millivolts; µV, microvolts; Co, controls.

### MRN Perfusion Parameters

No significant differences were found for perfusion parameters *K* ^trans,^ v_p_, and v_e_ between T2D patients and the control group. ANOVA found lower *K* ^trans^ values in patients with DN compared to T2D patients without DN (0.037 ± 0.010 vs. 0.044 ± 0.010; p=0.042) but not compared to controls (0.037 ± 0.010 vs. 0.035 ± 0.011; p=0.882). In patients with T2D and in controls, *K* ^trans^ was strongly correlated with v_e_ (r=0.75; p<0.001, and r=0.76; p=0.007, respectively). A summary of all correlations of perfusion parameters in T2D patients is provided in **Table 3**.

**Table 3:** Correlations of MRN perfusion parameters with demographic, serological, clinical, and electrophysiological data.

	K^trans^ (min^-1^) T2D		v_p_ (%) T2D		v_e_ (%) T2D		K^trans^ (min^-1^) Co		v_p_ (%) Co		v_e_ (%) Co	
	r	p	r	p	r	p	r	p	r	p	r	p
**K^trans^ (min^-1^)**			0.01	0.935	0.75	<0.001			-0.35	0.292	0.76	0.007
**v_p_ (%)**	-0.10	0.533			0.36	0.015	-0.35	0.292			0.08	0.818
**v_e_ (%)**	0.64	<0.001	0.36	0.015			0.76	0.007	0.08	0.818		
**Age (years)**	-0.24	0.120	-0.34	0.023	-0.34	0.022	0.13	0.706	0.17	0.622	0.39	0.232
**Diabetes duration (years)**	0.05	0.790	0.20	0.249	0.00	0.998	n.a.	n.a.	n.a.	n.a.	n.a.	n.a.
**Gender**	0.15	0.332	-0.13	0.385	0.16	0.300	0.04	0.915	0.21	0.542	-0.11	0.754
**BMI (kg/m^2^)**	0.55	<0.001	0.31	0.038	0.47	0.001	0.74	0.009	-0.29	0.393	0.30	0.369
**hsTNT (pg/mL)**	-0.38	0.012	-0.16	0.294	-0.30	0.048	0.39	0.241	-0.20	0.558	0.31	0.353
**proBNP (pg/mL)**	-0.09	0.569	-0.17	0.280	-0.05	0.732	0.03	0.931	-0.18	0.602	0.21	0.529
**HbA1c %**	-0.11	0.464	0.17	0.256	-0.01	0.953	0.35	0.298	-0.02	0.950	0.31	0.351
**GFR (ml/min)**	-0.19	0.242	0.09	0.594	-0.09	0.586	-0.11	0.754	0.07	0.835	0.03	0.920
**NDS**	-0.19	0.240	-0.01	0.948	-0.29	0.069	0.67	0.023	-0.24	0.468	0.71	0.014
**NSS**	-0.19	0.233	-0.09	0.578	-0.20	0.204	0.20	0.548	0.01	0.970	0.37	0.260
**Sural nerve NCV (m/s)**	0.45	0.030	-0.23	0.283	0.22	0.314	0.35	0.293	0.17	0.616	0.25	0.457
**Sural nerve SNAP (µV)**	0.32	0.095	-0.12	0.548	0.36	0.058	0.12	0.726	0.12	0.733	-0.24	0.482
**Peroneal NCV (m/s)**	0.49	0.002	-0.02	0.930	0.39	0.014	0.05	0.882	0.03	0.929	-0.33	0.317
**Peroneal CMAP (mV)**	0.27	0.099	0.11	0.494	0.23	0.161	0.62	0.042	-0.58	0.061	0.10	0.760
**Peroneal DML (ms)**	-0.18	0.261	-0.15	0.355	-0.48	0.002	-0.13	0.711	-0.31	0.354	-0.13	0.703
**Tibial NCV (m/s)**	0.42	0.008	-0.123	0.650	0.40	0.014	0.07	0.842	0.44	0.172	-0.02	0.949
**Tibial CMAP (mV)**	0.14	0.401	0.08	0.640	0.21	0.214	-0.16	0.638	0.35	0.286	-0.06	0.870
**Tibial DML (ms)**	-0.33	0.044	-0.24	0.156	-0.48	0.003	-0.12	0.724	-0.27	0.414	-0.05	0.893

K^trans^, constant of permeability; v_p_, plasma volume fraction; v_e_, extracellular extravascular volume fraction; BMI, body-mass index; hsTNT, high sensitivity troponin T; proBNP, pro brain binatriuretic peptide; n.a., not applicable; NDS, neuropathy disability score; NSS, neuropathy severity scale; NCV, nerve comnduction velocity; CMAP, compound motor action potential; SNAP, sensory nerve action potential; m/s, meters per second; ms, miliseconds; mV, millivolts; µV, microvolts.

### Correlation of Perfusion Parameters With Demographic Data

In patients with T2D and controls, *K* ^trans^ correlated positively with the BMI. Another correlation was found between v_e_ and BMI in patients with T2D. Parameter v_p_ was negatively correlated with age, while no such correlation was found for *K* ^trans^ or v_e_.

### Correlation of Perfusion Parameters With Serological, Clinical, and Electrophysiological Data

In T2D patients, *K* ^trans^ was positively correlated with NCVs of tibial, peroneal, and sural nerves. Parameter v_e_ was positively correlated with tibial and peroneal NCVs and DMLs. Correlations of *K* ^trans^ with tibial and peroneal NCVs are illustrated in **Figures 2C, D**. *K* ^trans^ and v_e_ were negatively correlated with hsTNT (**Figure 2E**). In a partial correlation analysis, double-controlled for confounding variables age and BMI, correlations remained significant between *K* ^trans^ and hsTNT (r=-0.42; p=0.005) and between v_e_ and hsTNT (r=0.33; p=0.034). No such correlations were found for *K* ^trans^ and v_e_ with proBNP, HbA1c, or the glomerular filtration rate. No correlations were found for v_p_ with hsTNT or electrophysiological parameters. A detailed summary of all correlations of MRN perfusion parameters with clinical and electrophysiological data in T2D patients and controls is provided in **Table 3**. Using a modified classification score for diabetic neuropathy severity based on electrophysiological parameters as proposed in (33), we allocated a score of 1 to patients with a sural nerve SNAP amplitude ≥ 5 µV, and a score of 2, 3, and 4 to patients with a sural nerve SNAP amplitude < 5 µV and a tibial nerve CMAP amplitude ≥ 5 mV, ≥ 2 mV and ≤ 5 mV, and < 2 mV, respectively, where neuropathy severity increases with score value. We subsequently found significant negative correlations between neuropathy severity score value and *K* ^trans^ (r=-0.41; p=0.007), and v_e_ (r=-0.37; p=0.017), indicating that neuropathy severity is associated with reduced nerve perfusion in agreement with previous animal experimental studies, possibly due to the development of abnormal microvasculature and capillary dysfunction (34, 35).

## Discussion

This study used DCE 3T MRN to investigate potential associations of peripheral nerve perfusion with cardiac biomarkers hsTNT and proBNP in patients with T2D. The main findings were (i) in T2D patients, hsTNT was negatively correlated with *K* ^trans^ and v_e_, while no such correlation was found for proBNP; (ii) in T2D, hsTNT, *K* ^trans^, and v_e_ were correlated with electrophysiological parameters and an electrophysiology-based neuropathy severity score; and (iii) hsTNT was increased while *K* ^trans^ and v_e_ were decreased in DN patients compared to patients without DN.

The results of this study confirm the hypothesis that hsTNT codifies parameters of nerve perfusion in patients with T2D (9, 12, 36). Specifically, the correlation of hsTNT with *K* ^trans^ suggests that an increase in hsTNT is associated with a decrease in capillary permeability of peripheral nerves. The correlations of *K* ^trans^ with v_e_ and between hsTNT and v_e_ further indicate that a decrease in nerve capillary permeability accompanied by elevated hsTNT levels is associated with a reduction of the extracapillary extracellular volume (EEV) fraction, which may ultimately result in nerve ischemia and demyelination. This assumption is further supported by the finding that hsTNT was negatively correlated with nerve conduction velocities of tibial and peroneal nerves, while *K* ^trans^, and v_e_ were positively correlated with nerve conduction velocities. Since a decrease in nerve conduction velocity is generally assumed to represent myelin damage (37), these correlations indicate that a decrease in capillary permeability and EEV fraction result in demyelination. In addition, the finding that there were no correlations for v_p_ with hsTNT or any of the acquired electrophysiological parameters further implies that there is no relevant impact of the capillary plasma volume fraction on structural nerve damage in the examined patients. The absence of correlations between proBNP and any of the acquired clinical and serological parameters in T2D patients is of importance for an understanding of the origin of elevated hsTNT levels in patients with T2D, meaning, that while there was a correlation of hsTNT and proBNP in healthy controls, no such correlation was found in the T2D group. This finding is of particular interest since it indicates that the well-established correlation of hsTNT and proBNP (38) does not apply in the T2D group. Since proBNP is an indicator for myocardial insufficiency, the lack of a correlation between hsTNT and proBNP indicates that elevated hsTNT levels in T2D patients and correlations of MRN perfusion parameters with hsTNT are not the consequence of myocardial insufficiency. Instead, our results are in line with previous studies suggesting that hsTNT represents myocardial damage due to microangiopathy that affects different organs in patients with T2D (9, 12). It remains to be determined, how much hyperglycemia or other metabolic factors, such as dyslipidemia, contribute to these microangiopathic changes (39–41).

One may of course argue, that hsTNT showed positive correlations with age while *K* ^trans^ showed positive correlations with BMI, therefore, the findings of this study only represent age- and obesity-related changes of perfusion in peripheral nerves. It should be considered, however, that negative correlations of *K* ^trans^ and v_e_ with hsTNT remained significant in a partial correlation analysis which was controlled for both age and BMI as confounding variables.

This study only found differences in perfusion parameters *K* ^trans^ and ve between T2D patients with and without DN, while no such difference was found between controls and T2D patients with DN. This is in line with previous studies on animal models for diabetes that found an increased vascular permeability in nerves of diabetic rats without diabetic neuropathy, supposedly due to an increased permeability of the basement membrane in Schwann cells and a reduction of nerve permeability in rats with DN compared to rats without DN, supposedly due to microangiopathy (42, 43).

This study is limited by the fact that only patients without an impairment of renal function were included due to the administration of MRI contrast agent. Thus, we cannot draw conclusions on the impact of hsTNT levels on nerve perfusion in patients with impaired renal function, since hsTNT is usually elevated in those patients due to renal elimination. Another limitation is the cross-sectional nature of the study which does not allow any conclusions on the predictive value of hsTNT for the progression of DN. The study is further limited by the sample size of T2D patients and controls, which cannot rule out all potential confounders for the observed differences and correlations. It should be considered, however, that patients and controls were matched for age, BMI, and renal function to minimize confounding and that correlations between hsTNT and perfusion parameters remained stable in a double-controlled partial correlation analysis.

In summary, this study found correlations between hsTNT and parameters of nerve perfusion obtained from 3T DCE MRN. The results indicate that hsTNT codifies a decrease in capillary permeability of peripheral nerves which is associated with a decrease in extravascular extracellular volume that ultimately causes demyelination as a result of nerve ischemia. Further longitudinal studies on the predictive value of hsTNT for the progression of DN, including the effects of age and BMI on nerve perfusion, are warranted.

## Data Availability Statement

The raw data supporting the conclusions of this article will be made available by the authors, without undue reservation.

## Ethics Statement

The studies involving human participants were reviewed and approved by Ethikkommission der Medizinischen Fakultät HeidelbergAlte Glockengießerei 11/169115 Heidelberg. The patients/participants provided their written informed consent to participate in this study.

## Author Contributions

JMEJ Study design and coordination, organization of participants, collection of MR data, image segmentation, data analysis and interpretation, literature search, writing of manuscript, arrangement of figures. CM: Collection of MR data, data analysis and interpretation, literature search, writing of manuscript, arrangement of figures. ZK: Collection of clinical, electrophysiological, and serological data, organization of participants; LS: Collection of clinical, electrophysiological, and serological data, organization of participants; AJ: organization of participants, collection of MR data, data analysis; SH: Conception of MRN sequence protocol; PN: Study design and coordination; MB: Study design and coordination, development of MR sequence protocol, writing of manuscript; SK: Development of clinical and electrophysiological study protocol, collection of clinical, electrophysiological, and serological data; FTK Study design and coordination, programming of image analysis tools, image segmentation, data analysis and interpretation, literature search, writing of manuscript, arrangement of figures. All authors contributed to the article and approved the submitted version.

## Funding

The German Research Foundation (DFG, SFB 1158), the Else Kröner-Fresenius-Stiftung (EKFS), and the International Foundation for Research in Paraplegia (Grant No. P179) provided financial support for personnel expenditure, MR imaging costs, and the technical equipment costs required for electrophysiological and serological analysis. The DFG, the EKFS, and the IRP had no influence on the study design, collection, and analysis of data or on the writing of the article.

## Conflict of Interest

JJ received grants from the German Research Foundation (SFB 1158), the Else Kröner-Fresenius-Stiftung, and from the International Foundation for Research in Paraplegia (Grant No. P179). ZK received grants from the Deutsches Zentrum für Diabetesforschung (DZD) e.V. SH received a grant from the Dietmar Hopp foundation and the German Research Council (DFG, SFB 1118). MB received grants and personal fees from Codman, Guerbet, Bayer and Novartis, personal fees from Roche, Teva, Springer, Boehringer, Grifols, Braun and grants from the European Union, Siemens, the Dietmar Hopp foundation, Stryker and the German Research Council (DFG, SFB 1118 and 1158). FK was supported by the German Research Foundation (KU 3555/1-1), the Hoffmann-Klose foundation and a research grant from Heidelberg University Hospital.

The remaining authors declare that the research was conducted in the absence of any commercial or financial relationships that could be construed as a potential conflict of interest.

## Publisher’s Note

All claims expressed in this article are solely those of the authors and do not necessarily represent those of their affiliated organizations, or those of the publisher, the editors and the reviewers. Any product that may be evaluated in this article, or claim that may be made by its manufacturer, is not guaranteed or endorsed by the publisher.
